# Circumventricular Organ Apelin Receptor Knockdown Decreases Blood Pressure and Sympathetic Drive Responses in the Spontaneously Hypertensive Rat

**DOI:** 10.3389/fphys.2021.711041

**Published:** 2021-08-04

**Authors:** Philip R. Griffiths, Stephen J. Lolait, Julian F. R. Paton, Anne-Marie O’Carroll

**Affiliations:** ^1^Faculty of Health Sciences, Bristol Medical School, University of Bristol, Bristol, United Kingdom; ^2^Department of Physiology, Faculty of Medical and Health Sciences, University of Auckland, Auckland, New Zealand; ^3^Faculty of Biomedical Sciences, School of Physiology, Pharmacology and Neuroscience, University of Bristol, Bristol, United Kingdom

**Keywords:** apelin, apelin receptor (APJ), circumventricular organs (CVOs), hypertension, spontaneously hypertensive rat (SHR)

## Abstract

The central site(s) mediating the cardiovascular actions of the apelin-apelin receptor (APJ) system remains a major question. We hypothesized that the sensory circumventricular organs (CVOs), interfacing between the circulation and deeper brain structures, are sites where circulating apelin acts as a signal in the central nervous system to decrease blood pressure (BP). We show that APJ gene (*aplnr*) expression was elevated in the CVOs of spontaneously hypertensive rats (SHRs) compared to normotensive Wistar Kyoto (WKY) controls, and that there was a greater mean arterial BP (MABP) decrease following microinjection of [Pyr^1^]apelin-13 to the CVOs of SHRs compared to WKY rats. Lentiviral APJ-specific-shRNA (LV-APJ-shRNA) was used to knockdown *aplnr* expression, both collectively in three CVOs and discretely in individual CVOs, of rats implanted with radiotelemeters to measure arterial pressure. LV-APJ-shRNA-injection decreased *aplnr* expression in the CVOs and abolished MABP responses to microinjection of [Pyr^1^]apelin-13. Chronic knockdown of *aplnr* in any of the CVOs, collectively or individually, did not affect basal MABP in SHR or WKY rats. Moreover, knockdown of *aplnr* in any of the CVOs individually did not affect the depressor response to systemic [Pyr^1^]apelin-13. By contrast, multiple knockdown of *aplnr* in the three CVOs reduced acute cardiovascular responses to peripheral [Pyr^1^]apelin-13 administration in SHR but not WKY rats. These results suggest that endogenous APJ activity in the CVOs has no effect on basal BP but that functional APJ in the CVOs is required for an intact cardiovascular response to peripherally administered apelin in the SHR.

## Introduction

Dysfunctions in mechanisms that regulate blood pressure (BP) participate in the development of hypertension that is mediated, in part, by activation of the sympathetic nervous system (SNS) (Zubcevic et al., 2011; Fisher and Paton, 2012) or by renal control of peripheral vascular resistance and body fluid volume (Wadei and Textor, 2012). A significant percentage of patients with essential hypertension, that accounts for 95% of all cases of hypertension (Carretero and Oparil, 2000), exhibit chronic sympathetic hyperactivity, but the causative mechanisms remain unclear.

The neuropeptide apelin (gene name *apln*) has well-established effects on cardiovascular and BP regulation via central and peripheral targets (O’Carroll et al., 2013) and mediates its effects via activation of the apelin receptor APJ (O’Dowd et al., 1993) (gene name *aplnr*). *Apln* encodes a 77-amino acid precursor (Tatemoto et al., 1998) that is enzymatically cleaved into several bioactive fragments, e.g., apelin-36, apelin-13, and subsequently processed into [Pyr^1^]apelin-13, which is the most abundant apelin isoform in the cardiovascular system (Maguire et al., 2009). APJ has highest homology to the angiotensin II receptor type AT1 and has been shown to antagonize the pressor effects of angiotensin II (Ang II) (Ishida et al., 2004; Siddiquee et al., 2013) by up-regulation of angiotensin-converting enzyme 2 (ACE2) and suppression of Ang II signaling (Sato et al., 2019). APJ and apelin are known key regulators of responses to multiple homeostatic perturbations including cardiovascular (Lee et al., 2000; Reaux et al., 2001; Seyedabadi et al., 2002; Ishida et al., 2004; Kuba et al., 2007; Kang et al., 2013; Wang et al., 2015; Yang et al., 2017), fluid (O’Carroll and Lolait, 2003), and food intake regulation (Reaux-Le Goazigo et al., 2011). The apelinergic system is implicated in cardiovascular and metabolic disease etiology and may act to mitigate the pathogenesis of heart failure and hypertension (Barnes et al., 2010) and of obesity-induced disorders that are common predisposing factors in hypertension (Dray et al., 2008; Sawane et al., 2013; O’Harte et al., 2018; Parthsarathy et al., 2018). Apelin and APJ mRNA and protein are expressed in the central nervous system (CNS), in regions such as the hypothalamic paraventricular nucleus (PVN) and the rostral ventrolateral medulla (RVLM) that participate in the control of BP, and also in a variety of peripheral organs including the pituitary gland, heart, adipose tissue, kidney, and gastrointestinal tract (O’Carroll et al., 2013). *Aplnr* expression in the PVN is up-regulated in response to acute and repeated stress (O’Carroll et al., 2003), while expression of *apln* is up-regulated in cardiac myocytes by hypoxia (Ronkainen et al., 2007). Insulin elevates both *aplnr* and *apln* expression, and apelin production, in adipose tissue (Sörhede Winzell et al., 2005; Dray et al., 2010). Furthermore, plasma apelin levels are increased by glucose administration (Soriguer et al., 2009) and in certain conditions such as heart failure with chronic systemic hypoxia (Ferdinal et al., 2019), and decreased by osmotic stimuli (De Mota et al., 2004). Plasma apelin concentration is also increased in insulin resistant subjects (Li et al., 2006), in type 2 diabetic (T2D) patients (Cavallo et al., 2012), and in obese individuals (Boucher et al., 2005). The peripheral apelinergic system appears to be down-regulated in hypertensive disease, with changes in the levels of both immunoreactive (ir)-APJ and/or *apln*/apelin in human (Sonmez et al., 2010; Chandra et al., 2011; Zhu et al., 2013) and some rodent hypertension models (Zhang et al., 2006).

The cardiovascular effects of apelin differ depending on whether it is administered centrally or peripherally. Apelin activates pressor mechanisms in the brain, where direct injection into the PVN or RVLM of rats increases BP (Seyedabadi et al., 2002; Zhang et al., 2009, 2014; Griffiths et al., 2018), while the overall effect of peripherally administered apelin, which may be acting via APJ present at peripheral and/or central sites, decreases BP in multiple animal models (Lee et al., 2000; Reaux et al., 2001; Mitra et al., 2006; Siddiquee et al., 2013), with the magnitude of the depressor effect being enhanced in hypertensive animals such as the spontaneously hypertensive rat (SHR) (Lee et al., 2005). Interestingly, APJ and apelin mRNAs are expressed within the sensory circumventricular organs (CVOs) (Hatzelmann et al., 2009; Griffiths et al., 2020), comprised of the subfornical organ (SFO), organum vasculosum of lamina terminalis (OVLT), and the area postrema (AP). These anatomical sites, lacking a blood-brain barrier (BBB), have an important role in cardiovascular regulation (Cottrell and Ferguson, 2004); they act as anatomical conduits to major sympathetic regulatory centers (McKinley et al., 2001) and display a variety of efferent and afferent inter-neuronal connections that may contribute to a composite cardiovascular response (Swanson and Kuypers, 1980; Hasser et al., 2000; Cottrell and Ferguson, 2004). CVO neurons acts as sensors for circulating neuropeptides such as Ang II (Sunn et al., 2003), leptin (Smith et al., 2009), and vasopressin (Smith and Ferguson, 1997), indicating an established role for these organs as sites for monitoring peripheral signals. Microinjection of apelin into the SFO decreases BP (Dai et al., 2013), a cardiovascular response similar to that seen with peripheral administration of apelin. Additionally, we have shown increased *aplnr* expression in the SFO of SHRs [an established and well-characterized model of hypertension (Trippodo and Frohlich, 1981)] in comparison to control Wistar Kyoto (WKY) rats (Griffiths et al., 2020), providing evidence of a likely association for SFO APJ in the pathogenesis of hypertension in the SHR. The role of the apelinergic system in CVO functions other than electrophysiological and cardiovascular responses, such as sodium and water balance, is not known.

While the sites by which circulating apelin exerts its central effects to modulate BP are undetermined, the sensory CVOs, in addition to the PVN and RVLM, may mediate the cardiovascular actions of apelin. We hypothesized that the sensory CVOs act as a link integrating the effects of circulating and central apelin on BP regulation. In this study we used RNA interference (RNAi) to examine whether down-regulation of *aplnr* expression in individual sensory CVOs, or alternatively simultaneous down-regulation of *aplnr* expression in three sensory CVOs in the same animal, blocks the effects of central and/or peripheral circulating apelin mediating cardiovascular responses in normotensive WKY and hypertensive SHR rats.

## Materials and Methods

### Ethical Approval

All experiments were approved by the University of Bristol Animal Welfare and Ethical Review Body and performed in strict accordance with U.K. Home Office regulations [Animals (Scientific Procedures) Act (1986)].

### Animals

Adult male (∼250 g) Wistar (*n* = 18, Charles River, United Kingdom), Wistar Kyoto (WKY; *n* = 34; Envigo, United Kingdom) and adult spontaneously hypertensive rats (SHR; *n* = 34; Envigo, United Kingdom) were housed at a constant temperature (21 ± 2°C) and humidity on a 14:10 h light:dark cycle. Access to standard laboratory chow and water was provided *ad libitum*.

### Acute Exogenous [Pyr^1^]apelin-13 Microinjection Into CVOs

**FIGURE 1:**
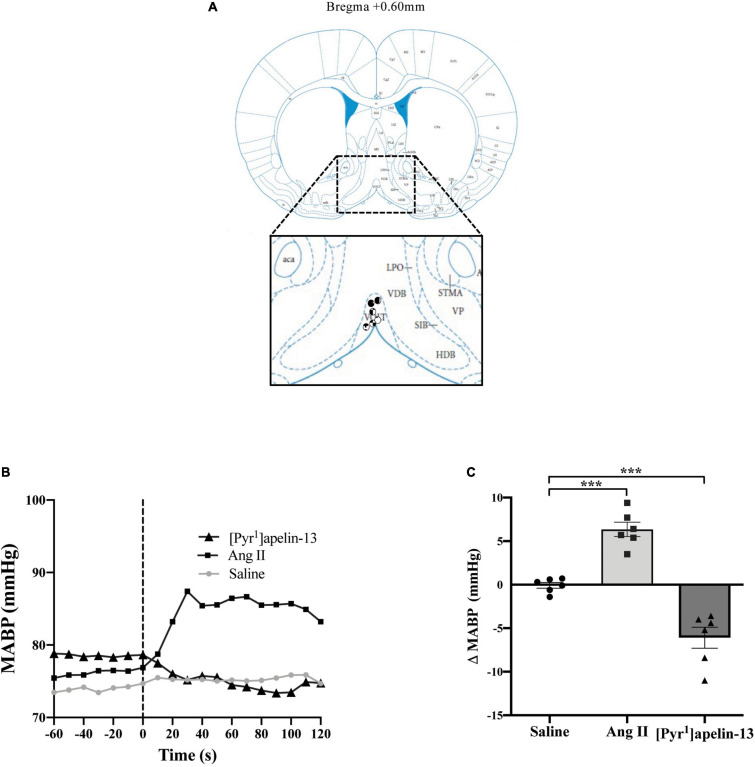
Central apelin microinjection into the OVLT decreases arterial blood pressure. **(A)** Schematic illustrating the localization of injection sites (symbols indicate injection sites) in the OVLT, representative of sections (level relative to bregma indicated) from each animal group, determined by examination of the deposition of dye in the brain stem of animals. In inset: aca, anterior commissure, anterior part; AcbC, accumbens nucleus, core; HDB, nucleus of the horizontal limb of the diagonal band; LPO, lateral preoptic area; SIB, substantia innominata, basal part; STMA, bed nucleus of the stria terminalis, medial division, anterior part; VOLT, vascular organ of the lamina terminalis (or OVLT); VDB, nucleus of the vertical limb of the diagonal band; VP, ventral pallidum (adapted from Paxinos and Watson, 2005; Figure 28). **(B)** Representative raw traces, binned over 1 s, showing the BP response in single animals following microinjection of [Pyr^1^]apelin-13 (200 pmol 100 nl^–1^), angiotensin II (Ang II) (100 pmol 100 nl^–1^), and saline to the OVLT. The dotted line indicates the microinjection of compound. **(C)** Change in MABP following microinjection of saline, Ang II (100 pmol 100 nl^–1^) and [Pyr^1^]apelin-13 (200 pmol 100 nl^–1^) to the OVLT of Wistar rats (*n* = 6). Data is mean ± SEM with individual responses. Data analyzed with 1-way ANOVA followed by Tukey’s Multiple Comparison test. ***P < 0.001.

**FIGURE 2:**
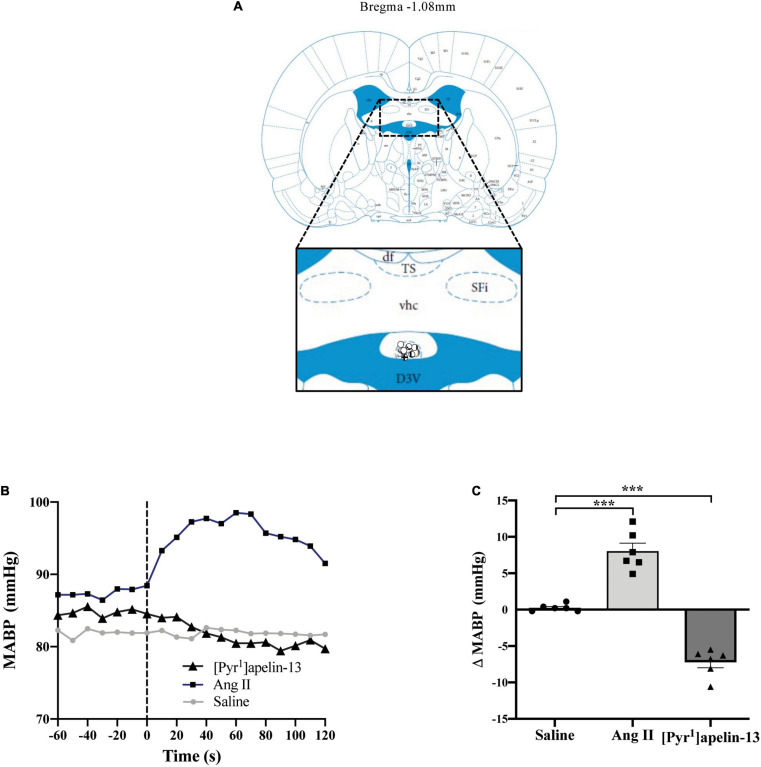
Central apelin microinjection into the SFO decreases arterial blood pressure. **(A)** Schematic illustrating the localization of injection sites (symbols indicate injection sites) in the SFO, representative of sections (level relative to bregma indicated) from each animal group, determined by examination of the deposition of dye in the brain stem of animals. In inset: D3V, dorsal 3rd ventricle; df, dorsal fornix; Sfi, septofimbrial nucleus; SFO, subfornical organ; TS, triangular septal nucleus; vhc, ventral hippocampal commissure (adapted from Paxinos and Watson, 2005; Figure 42). **(B)** Representative raw traces, binned over 1 s, showing the BP response in single animals following microinjection of [Pyr^1^]apelin-13 (200 pmol 100 nl^–1^), angiotensin II (Ang II) (100 pmol 100 nl^–1^), and saline to the SFO. The dotted line indicates the microinjection of compound. **(C)** Change in MABP following microinjection of saline, Ang II (100 pmol 100 nl^–1^) and [Pyr^1^]apelin-13 (200 pmol 100 nl^–1^) to the SFO of Wistar rats (*n* = 6). Data is mean ± SEM with individual responses. Data analyzed with 1-way ANOVA followed by Tukey’s Multiple Comparison test. ***P < 0.001.

**FIGURE 3:**
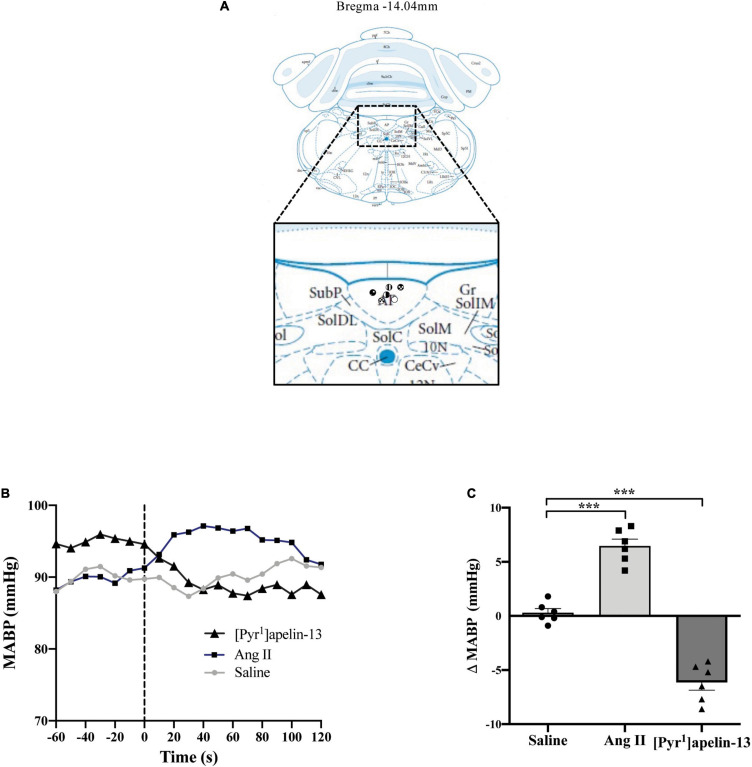
Central apelin microinjection into the AP decreases arterial blood pressure. **(A)** Schematic illustrating the localization of injection sites (symbols indicate injection sites) in the AP, representative of sections (level relative to bregma indicated) from each animal group, determined by examination of the deposition of dye in the brain stem of animals. In inset: 10 N, dorsal motor nucleus of vagus; AP, area postrema; CC, central canal; CeCV, central cervical nucleus of the spinal cord; Gr, gracile nucleus; SolC, nucleus of the solitary tract, commissural part; SolDL, solitary nucleus, dorsolateral part; SolIM, nucleus of the solitary tract, intermediate part; SolM, nucleus of the solitary tract, medial part; SubP, subpostrema area (adapted from Paxinos and Watson, 2005; Figure 150). **(B)** Representative raw traces, binned over 1 s, showing the BP response in single animals following microinjection of [Pyr^1^]apelin-13 (200 pmol 100 nl^–1^), angiotensin II (Ang II) (100 pmol 100 nl^–1^), and saline to the AP. The dotted line indicates the microinjection of compound. **(C)** Change in MABP following microinjection of saline, angiotensin II (Ang II 100 pmol, 100 nl^–1^) and [Pyr^1^]apelin-13 (200 pmol 100 nl^–1^) to the AP of Wistar rats (*n* = 6). Data is mean ± SEM with individual responses. Data analyzed with 1-way ANOVA followed by Tukey’s Multiple Comparison test. ***P < 0.001.

To establish CVO co-ordinates and for preliminary investigation of [Pyr^1^]apelin-13 pharmacology in each CVO, Wistar rats were anesthetized by intraperitoneal (i.p.) injection of sodium pentobarbital (50 mg/kg). All surgical procedures were performed under aseptic conditions under a surgical plane of anesthesia as indicated by the absence of withdrawal reflex to hindpaw pinch, which was monitored throughout the procedure and additional doses of anesthetic given as necessary. BP was monitored via a heparin-saline (1 U/ml) filled catheter (Micro-Renathane tubing, 0.8 mm inner diameter, Braintree Scientific) implanted in the left or right femoral artery and connected to a pressure transducer (BD DTX Plus, Southwest Medical, Bristol, United Kingdom). For injections to the SFO and OVLT the head was secured in a stereotaxic frame with the head held level (confirmed by bregma and lambda having the same depth coordinate). For injections to the AP the head was flexed down (nose bar at −19 mm), and the atlanto-occipital membrane pierced to allow visualization, with the aid of a binocular surgical microscope (Leica M651, United Kingdom), of the AP as a highly vascularized structure above the Obex on the midline dorsal surface of the medulla oblongata. The CVOs were targeted using previously established stereotaxic co-ordinates: OVLT: 0.4 mm rostral to bregma, 0 mm lateral to midline, 8.0 mm below dura; SFO: 1.3 mm caudal to bregma, midline, 4.5 mm below dura [differences between these levels and those depicted in the representative atlas sections in Figures 1–3 are accounted for by the age difference of our rats (∼250 g) and those (∼290 g) used to construct the reference atlas]. Injections to all structures were made using single-barreled micropipettes (1–5 μl pre-calibrated microcapillary tube, Sigma Aldrich, United Kingdom) using a binocular surgical microscope. Microinjection of Ang II (100 pmol 100 nl^–1^; Tocris Bioscience, United Kingdom) was used to functionally confirm the position of the pipette within the CVOs by recording a rise in arterial pressure (Reaux et al., 2001). The injection volume was 100 nl. Subsequent microinjections of [Pyr^1^]apelin-13 or vehicle were made at the same stereotaxic coordinates that elicited the largest Ang II pressor response (∼8–10 mmHg). The effective dose of [Pyr^1^]apelin-13 was determined by a dose response course (67, 200, 600, and 1800 pmol 100 nl^–1^; *n* = 6/group) measuring [Pyr^1^]apelin-13-induced cardiovascular effects in the CVOs and was found to be maximally effective at 200 pmol 100 nl^–1^ in each CVO. A subset of animals received a microinjection of Indian ink (100 nl, 1:10 dilution) at the end of the experiment to confirm the location of the stereotaxic coordinates used. After the final microinjection, rats were deeply anesthetized with sodium pentobarbital and euthanized by decapitation.

### Chronic Arterial Blood Pressure Measurement

#### Chronic Femoral Vein Cannulation

Intravenous (i.v.) cannulas were constructed and implanted as described previously (Griffiths et al., 2018) to allow i.v. administration of pharmacological compounds. Briefly, adult WKY (*n* = 34) and SHR (*n* = 34) rats were anesthetized with isoflurane (4% for induction and 2% for maintenance in O_2_) and provided with analgesia (Vetergesic; 0.8 mg kg^–1^). Cannulas were implanted in the left femoral vein, tunneled subcutaneously, and externalized between the scapulae. Following surgery, rats were housed singly in a soundproofed room. Cannulae were flushed with 0.5 ml heparinized saline (50 IU ml^–1^) daily for 1 week following surgery and then every other day for the remainder of the experiment.

#### Telemeter Implantation

A radiotelemetry system (TRM54P, Kaha Sciences, Auckland, New Zealand) was adopted to make chronic, continuous measurements of arterial pressure as we have reported previously (McBryde et al., 2013; Griffiths et al., 2018). Adult WKY (*n* = 34) and SHR (*n* = 34) rats underwent telemetry surgery 3 days following i.v. cannulation. Animals were allowed 6 days recovery following telemeter implantation before continuous recording of BP for 5 days before (baseline), and 18 days after (experimental), virus injection surgeries.

### Gene Transfer

#### Transfer of Viral Vectors Into the CVOs

The *aplnr*-specific (LV-APJ-shRNA) and non-targeting scrambled (LV-scr-shRNA) short hairpin RNAs (shRNAs), incorporating green fluorescent protein (GFP), were expressed in the lentiviral transfer vector pRRL.SIN.CPPT.CMV.GFP.WPRE (modified from Addgene plasmid 12252). Lentiviral particles (LV) were produced using packaging plasmids pMDLg/pRRE, pRSV-Rev and PMD2.G (Addgene) to a titer of LV-APJ-shRNA, 1.12 × 10^10^ pfu ml^–1^ and LV-scr-shRNA, 4.3 × 10^9^ pfu ml^–1^ as described previously (Griffiths et al., 2018). The effective time point after virus transfection into the CVOs was determined by a time course (18 and 25 days; *n* = 8/group) measuring loss of [Pyr^1^]apelin-13-induced cardiovascular effects in the CVOs and was found to be maximally effective at 18 days.

#### Intraparenchymal Injection of Lentivirus

Eleven days post telemeter implantation and after 5 days of baseline recording, WKY and SHR rats (*n* = 34/group) were randomly assigned to either the LV-APJ-shRNA or control LV-scr-shRNA lentiviral particles group. Animals were anesthetized with an intramuscular injection of ketamine (60 mg kg^–1^) and medetomidine (250 μg kg^–1^), analgesia administered (Vetergesic 0.8 mg kg^–1^), and microinjected either individually in one of the OVLT (*n* = 4/group), SFO (*n* = 6/group) or AP (*n* = 4/group), or in the three CVOs simultaneously (*n* = 3/group), with either LV-APJ-shRNA or LV-scr-shRNA (titre > 10^9^ transforming units/ml; 1 μl). The micropipette was advanced to the correct depth for the structure of interest (using the brain coordinates outlined above) and allowed to rest for 5 min prior to viral vector (1 μl) delivery over a period of 10 min. The pipette was then left *in situ* for a further 5 min to minimize back-tracking of the virus. Anesthesia was reversed by a subcutaneous injection of antisedan (1 mg kg^–1^). BP recording continued for 18 days (see data acquisition below).

### Terminal Experiment

#### Chronic *Aplnr* CVO Knockdown and Cardiovascular Responses to i.v. Administered Apelin

Eighteen days post virus injection(s), rats were anesthetized with sodium pentobarbital (50 mg kg^–1^ i.p.) and cardiovascular parameters monitored using the implanted telemeter. [Pyr^1^]apelin-13 (2 nmol/kg; 300 μl, i.v) and vehicle (saline, 300 μ l, i.v.) was administered to assess any changes in apelin-induced pressor responses. Following injection, BP was allowed to recover for 30 min.

#### Chronic *Aplnr* CVO Knockdown and Cardiovascular Responses to Central Microinjection of Apelin

To confirm that chronic knock-down of *aplnr* blocked mean arterial blood pressure (MABP) responses, a direct “challenge” microinjection of [Pyr^1^]apelin-13 (200 pmol 100 nl^–1^) to the OVLT, SFO, and/or AP, similar to that described previously for the RVLM (Griffiths et al., 2018), was carried out. BP was monitored via the implanted telemeter. CVO injections followed the same protocol described above. Rats were then euthanized using a guillotine. Brains were immediately frozen on powdered dry ice and stored at −80°C to preserve RNA integrity. The level of *aplnr* knockdown was confirmed by quantitative PCR (qPCR) (see below).

### RNA Extraction and cDNA Synthesis

Fresh, frozen sections (40 μm) were cut on a cryostat (CM3050 S, Leica Microsystems) and collected on the freezing plate. Every fifth section was stained with 0.1% toluidine blue to aid identification of the CVOs and samples were then collected using a 1 mm diameter micropunch (Fine Scientific Tools) and placed into separate 1.5 ml RNAse/DNAse-free tubes (Appleton Woods, United Kingdom) on dry ice and stored at −80°C, before RNA extraction. The protocols for RNA extraction and cDNA synthesis from brain punches have been described previously (Griffiths et al., 2018).

### Real Time qPCR Analysis

Primers used and the protocol followed are as described (Griffiths et al., 2018). qRT-PCR was carried out in duplicate using SYBR green (Thermo Fisher Scientific, United Kingdom) on an ABI StepOnePlus Sequence Detection System (ABI, Warrington, United Kingdom). All qRT-PCR reactions were followed by dissociation curve analysis. For relative quantification of gene expression the 2^–ΔΔCT^ method was employed (Livak and Schmittgen, 2001). The internal control genes used were the housekeeping genes ribosomal protein L19 (*Rpl19)* and glyceraldehyde 3-phosphate dehydrogenase (*Gapdh*).

### Data Analysis

BP waveforms were captured using a CED 1401 data capture system and Spike 2.7 software (CED, United Kingdom) sampled at 2 kHz. BP waveforms were processed offline using the HRBP (version 8) script for Spike 2 in order to extract systolic (SABP), diastolic (DABP) and MABP, pulse interval (PI), respiratory rate (RR), and heart rate (HR) waveforms. For acute experiments on exogenous [Pyr^1^]apelin-13 microinjection into Wistar rat CVOs using pressure transducers, representative MAPB readings are shown and data is presented as change in physiological variable by subtraction of the post-microinjection response from the pre-microinjection baseline for each compound. BP data are presented as mean ± SEM and tested for statistical significance using a repeated measures 1-way ANOVA followed by Tukey’s Multiple Comparison test using Graphpad Prism 8.0 software for Mac (Graphpad Software Inc., United States). Statistical significance for differences between groups was defined as *P* < 0.05.

For chronic experiments on *aplnr* CVO knockdown, baseline values of physiological variables were recorded and averaged over 5 days before virus injection surgery. In these studies, raw BP waveforms were recorded for the first 10 min of each hour and BP, HR, and RR data was recorded continuously online. Data were then recorded for a subsequent 18 days following virus injection. BP, HR and RR data were extracted offline following acute administration of [Pyr^1^]apelin-13 or Ang II. Spectral analysis of systolic BP was performed using HRV1 script for Spike 2 to calculate power in the very low frequency (VLF): 0–0.3 Hz; LF: 0.3–0.8 Hz and high frequency (HF): 0.8–3.3 Hz frequency bands (Parati et al., 1995; Japundžić-Žigon, 1998). The following settings were used to compute the power spectra: time constant for DC removal: ± 3 s; frequency range of spectra: 0–5.12 Hz; epoch duration: 25 s; FFT size: 128; window type: Hanning. While it has been established that LF-SBPV can be used as an indirect measure of vasomotor sympathetic tone (Waki et al., 2006; Lozić et al., 2014) there is still an ongoing debate regarding this use of LV-SBPV as a marker for estimating sympathetic activation. Therefore, to confirm that any changes in spectral analysis data between experimental groups was associated with change in sympathetic nervous activity, rats additionally were injected with hexamethonium (i.v.; 10 mg kg^–1^) 16 days after virus injection to *directly* assess the relative level of tone as indicated by the extent of the fall in arterial pressure. The change in BP following hexamethonium injection was calculated by subtracting MABP over the 10 s period following injection from a 10 s baseline period. Statistical analysis was carried out using Graphpad Prism 8.0 software (Graphpad Software Inc., United States). Statistical significance for differences between groups was defined as *P* < 0.05. Unless stated differently, statistical differences between two experimental groups were evaluated using independent-sample unpaired Student’s *t*-tests. Experimenters were not blinded to the conditions being analyzed.

## Results

### Central Apelin Microinjection Into the CVOs Decreases Arterial Blood Pressure

Schematics illustrating the localization of injection sites in the OVLT, SFO, and AP in coronal sections are shown in Figures 1A, 2A, 3A, respectively. Representative raw traces from single animals showing the BP response following microinjection of [Pyr^1^]apelin-13, Ang II, and saline to the OVLT, SFO, and AP are shown in Figures 1B, 2B, 3B, respectively. Microinjection of Ang II (100 pmol) into the OVLT, SFO, or AP, significantly increased MABP in the OVLT (Figure 1C), SFO (Figure 2C), and AP (Figure 3C) of Wistar rats, compared to saline-injected controls. Microinjection of [Pyr^1^]apelin-13 (200 pmol) into the OVLT, SFO, or AP decreased MABP (Figures 1C, 2C, 3C, respectively) compared to saline-injected controls.

### Peripheral Apelin Injection Decreases Arterial Blood Pressure in SHR and WKY Rats

**FIGURE 4:**
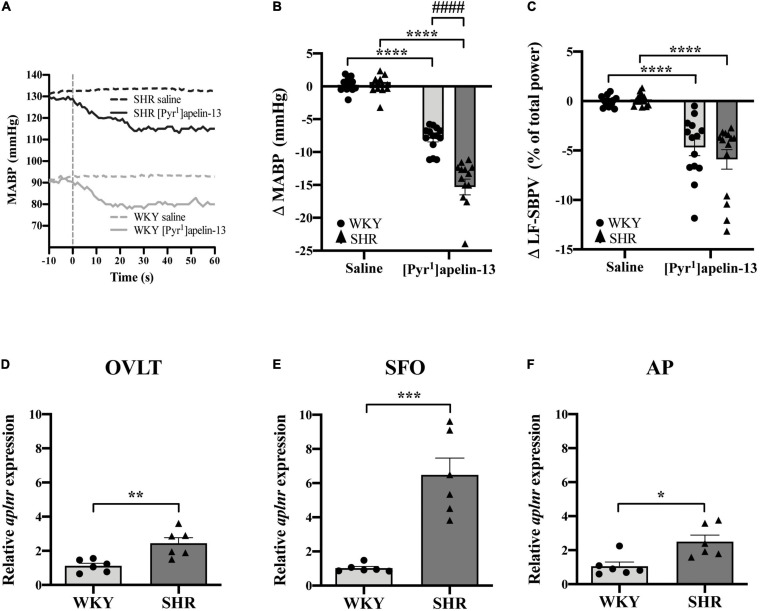
Peripheral [Pyr^1^]apelin-13 injection decreases arterial blood pressure and SBPV in SHR and WKY rats and *aplnr* expression is up-regulated in the CVOs. **(A)** Representative raw traces showing the BP response in single animals following peripheral i.v. injection (vertical dotted line) of [Pyr^1^]apelin-13 (2 mol/kg) and saline to WKY and SHR rats. Change in **(B)** MABP and **(C)** LF-SBPV following i.v. injection of saline and [Pyr^1^]apelin-13 (2 mol/kg) to WKY (light gray bar, circles) and SHR (dark gray bar, triangles) rats. Data is mean ± SEM with individual responses (*n* = 14/group). In **(B)** [Pyr^1^]apelin-13-injected WKY rats vs. [Pyr^1^]apelin-13-injected SHRs was analyzed with Student’s unpaired *t*-test, **^####^***P* < 0.0001. All other data in **(B,C)** was analyzed with Student’s paired *t*-tests, ****P < 0.0001. Relative expression of *aplnr* in **(D)** OVLT, **(E)** SFO and **(F)** AP micropunches from WKY (light gray bar, circles) and SHR (dark gray bar, triangles) rats. Data is mean ± SEM with individual responses (*n* = 6/group). Data analyzed with Student’s unpaired *t*-tests. *P < 0.05, **P < 0.01, ***P < 0.001.

Baseline physiological variables in awake normotensive WKY rats and hypertensive SHRs are summarized in Table 1. Baseline MAPB was 103 ± 3 vs. 138 ± 3 mmHg in WKY and SHR, respectively. Representative raw traces from single animals showing the change in MABP following i.v. injection of [Pyr^1^]apelin-13 in WKY rats and SHRs are shown in Figure 4A. Peripheral injection of [Pyr^1^]apelin-13 (2 nmol/kg; i.v. by femoral vein) in both WKY rats and SHRs decreased MABP and LF-SBPV (Figures 4B,C, respectively) compared to saline-injected controls. The decrease in MABP in response to i.v. [Pyr^1^]apelin-13 was significantly greater in SHR than in WKY rats (Figure 4B).

**TABLE 1:** Baseline physiological parameters measured prior to virus injection in WKY and SHR rats.

	WKY		SHR	
	LV-scr-shRNA	LV-APJ-shRNA	LV-scr-shRNA	LV-APJ-shRNA
DABP (mmHg)	83 ± 3	87 ± 5	106 ± 4	117 ± 3
SABP (mmHg)	127 ± 3	127 ± 5	169 ± 4	181 ± 3
MABP (mmHg)	103 ± 3	104 ± 5	138 ± 3	147 ± 2
PP (mmHg)	44 ± 1	44 ± 1	64 ± 1	64 ± 1
RR (breaths/min)	93 ± 1	93 ± 1	87 ± 1	88 ± 1
HR (bpm)	312 ± 5	314 ± 7	306 ± 3	308 ± 2
Weight (g)	245 ± 3	243 ± 3	268 ± 3	263 ± 2

*Data is mean ± SEM, n = 17/group.*

### *Aplnr* Expression Is Up-Regulated in OVLT, SFO, and AP of SHR

To examine *aplnr* expression in the CVOs from normotensive and hypertensive rats, expression levels in micropunches of CVOs from WKY rats and SHRs were quantified using qPCR. *Aplnr* transcript levels were higher in OVLT, SFO, and AP in SHRs in comparison with WKY control rats (Figures 4D–F, respectively).

### LV-APJ-shRNA-Injection in Individual CVOs in WKY and SHRs Decreases *Aplnr* Expression and Silences Cardiovascular Responses to Microinjection of [Pyr^1^]apelin-13

**FIGURE 5:**
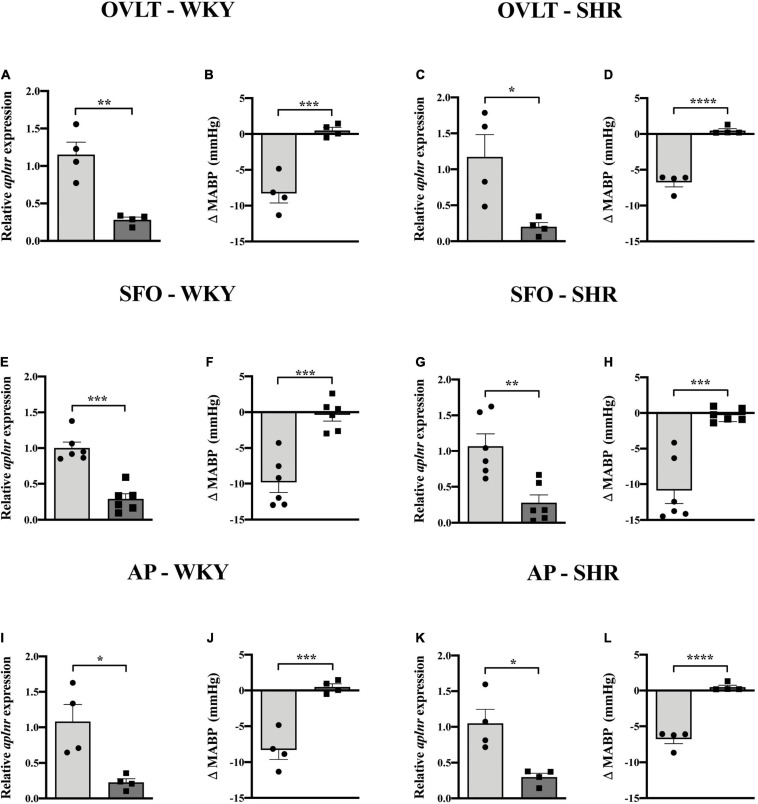
LV-APJ-shRNA-injection in individual CVOs in WKY and SHRs decreases *aplnr* expression and silences cardiovascular responses to microinjection of [Pyr^1^]apelin-13. Relative *aplnr* expression in micropunched tissue **(A,C,E,G,I,K)** and change in MABP following microinjection of [Pyr^1^]apelin-13 (200 pmol 100 nl^–1^) **(B,D,F,H,J,L)** 18 days following microinjection of either LV-scr-shRNA (light gray bar, circles) or LV-APJ-shRNA (dark gray bar, squares) to the OVLT of WKY **(A,B)** and SHR **(C,D)** rats (*n* = 4/group), to the SFO of WKY **(E,F)** and SHR **(G,H)** rats (*n* = 6/group), and to the AP of WKY **(I,J)** and SHR **(K,L)** rats (*n* = 4/group). Data is mean ± SEM with individual responses. Data analyzed with Student’s unpaired *t*-tests. *P < 0.05, **P < 0.01, ***P < 0.001, ****P < 0.0001.

Lentiviral vector-mediated gene delivery was used to knockdown *aplnr* expression in each of the OVLT, SFO, and AP individually in WKY rats and in SHRs. qPCR analysis of micropunches from the CVOs of animals microinjected with LV-APJ-shRNA confirmed successful knockdown of *aplnr* expression in the OVLT, SFO, or AP at day 18 post-virus injection in WKY rats (Figures 5A,E,I, respectively) and SHRs (Figures 5C,G,K, respectively) compared with those control animals injected with LV-scr-shRNA. The effectiveness of the LV-APJ-shRNA injection was additionally verified by measuring the cardiovascular responses of rats to a challenge microinjection of [Pyr^1^]apelin-13 individually into each of the OVLT, SFO or AP of anesthetized WKY and SHRs at day 18 post-virus injection. For each of the OVLT, SFO, and AP, microinjection of [Pyr^1^]apelin-13 decreased MABP in LV-scr-shRNA-injected-WKY rats (Figures 5B,F,J, respectively) and -SHRs (Figures 5D,H,L, respectively). Knockdown of *aplnr* in the individual CVOs by LV-APJ-shRNA-injection prevented this decrease in MABP in response to microinjection of [Pyr^1^]apelin-13 into the OVLT, SFO, or AP of WKY rats (Figures 5B,F,J, respectively), or SHRs (Figures 5D,H,L, respectively).

### Individual CVO-Targeted Chronic *Aplnr* Knockdown Does Not Block the Peripheral Depressor Effect of [Pyr^1^]apelin-13 in Normotensive or Hypertensive Rats

At any point during the 18-day monitoring period, no significant difference was seen in MABP or LF-SBPV between animals transduced individually in either the OVLT, SFO, or AP with either LV-APJ-shRNA or LV-scr-shRNA in WKY rats or SHRs (Table 2). Falls in MABP induced by i.v. hexamethonium injection were similar between all OVLT, SFO, and AP LV-APJ-shRNA- or LV-scr-shRNA-injected WKY rats or SHRs (Table 2).

**TABLE 2:** Individual OVLT-, SFO-, or AP-targeted knockdown of *aplnr* in WKY and SHR rats.

	WKY		SHR	
Targeted CVO	LV-scr-shRNA	LV-APJ shRNA	LV-scr-shRNA	LV-APJ shRNA
	**Time course Δ MABP (mmHg)**			
OVLT	−3.07 ± 2.65	1.68 ± 2.37	14.39 ± 7.54	16.26 ± 5.75
SFO	1.59 ± 1.44	−0.93 ± 1.13	1645 ± 3.01	10.72 ± 1.64
AP	2.28 ± 2.18	0.28 ± 3.33	9.94 ± 1.21	17.45 ± 2.24
	**Time course Δ LF-SBPV (% total power)**			
OVLT	−3.28 ± 0.88	−2.08 ± 0.60	−3.92 ± 0.70	−2.93 ± 1.92
SFO	−2.17 ± 0.95	−1.72 ± 0.53	−2.58 ± 1.51	−4.62 ± 1.58
AP	−1.81 ± 0.98	−2.16 ± 1.12	−2.82 ± 1.18	−2.83 ± 0.88
	**Effect of hexamethonium Δ MABP (mmHg)**			
OVLT	−15.27 ± 1.44	−14.95 ± 1.42	−20.63 ± 1.16	−23.54 ± 1.62
SFO	−17.96 ± 0.71	−18.14 ± 2.11	−18.80 ± 4.10	−17.60 ± 1.17
AP	−16.27 ± 1.75	−17.95 ± 2.89	−20.63 ± 1.16	−23.54 ± 1.62
	**Effect of i.v. [Pyr^1^]apelin-13 injection Δ MABP (mmHg)**			
OVLT	−7.85 ± 0.85	−8.24 ± 0.98	−16.24 ± 2.97	−14.29 ± 3.25
SFO	−9.13 ± 1.66	−9.72 ± 2.21	−17.96 ± 0.71	−18.14 ± 2.11
AP	−8.32 ± 0.97	−9.46 ± 1.71	−20.74 ± 4.78	−15.54 ± 4.43
	**Effect of i.v. [Pyr^1^]apelin-13 injection Δ LF-SBPV (% total power)**			
OVLT	−5.01 ± 0.97	−4.46 ± 1.41	−5.09 ± 1.54	−7.56 ± 0.97
SFO	−3.67 ± 1.69	−5.45 ± 1.57	−3.54 ± 0.13	−4.50 ± 1.10
AP	−5.89 ± 1.15	−5.40 ± 0.60	−8.74 ± 2.26	−5.61 ± 1.41

*Individual OVLT- (n = 4/group), SFO- (n = 6/group), or AP-targeted (n = 4/group) knockdown of aplnr in WKY or SHR rats had no effect on chronic MABP (mm Hg), chronic LF-SBPV (% total power), hexamethonium-induced falls in MABP (mm Hg), the peripheral depressor effect of [Pyr^1^]apelin-13 (mm Hg) or the peripheral-induced decrease in LF-SBPV (% total power). Data is mean ± SEM.*

At day 18 post-virus injection, all groups of individual CVO-targeted WKY and SHRs were injected i.v. with [Pyr^1^]apelin-13 or saline control and MABP recorded. The decrease in MABP observed following peripheral administration of [Pyr^1^]apelin-13 was maintained despite *aplnr* knockdown individually in the OVLT, SFO, or AP of WKY rats or SHRs (Table 2). Similarly, the decrease in LF-SBPV observed following peripheral administration of [Pyr^1^]apelin-13 was maintained despite *aplnr* knockdown individually in the OVLT, SFO, or AP of WKY rats or SHRs (Table 2).

### Multiple CVO-Targeted *Aplnr* Knockdown Does Not Block the Peripheral Depressor Effect of [Pyr^1^]apelin-13 in Normotensive Rats

**FIGURE 6:**
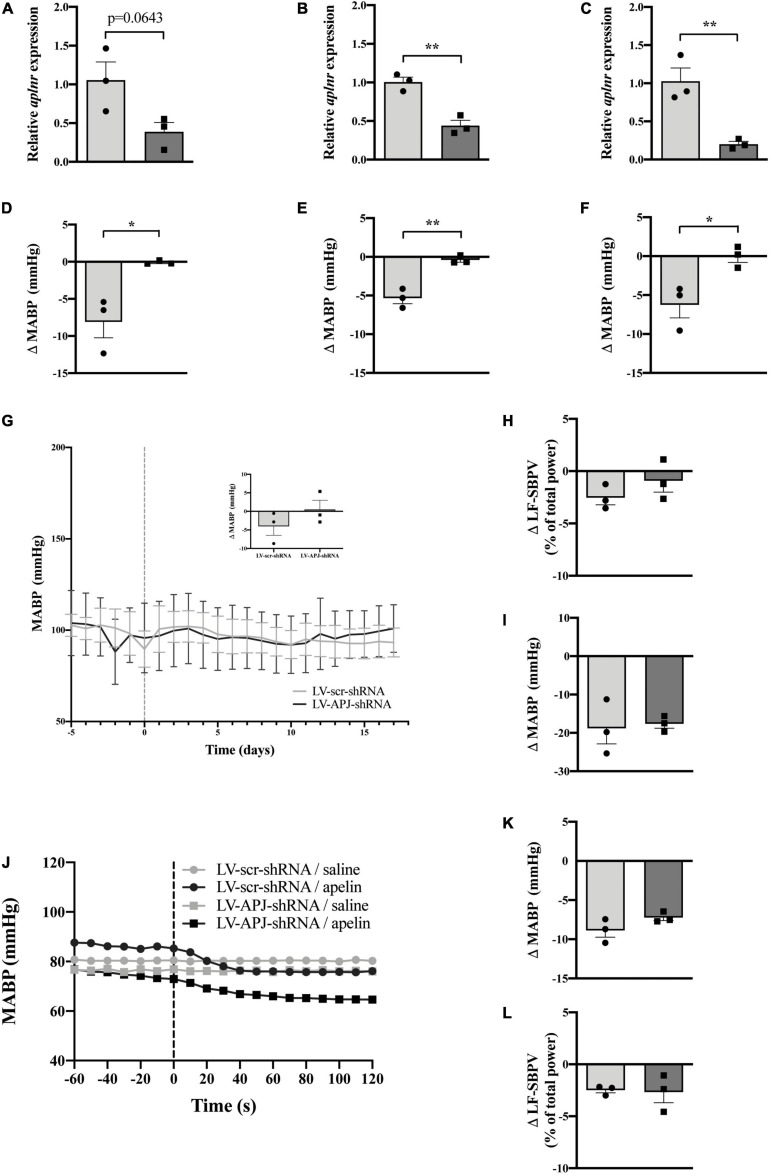
Simultaneous CVO-targeted *aplnr* knockdown does not block the peripheral depressor effect of [Pyr^1^]apelin-13 in WKY rats. Relative *aplnr* expression in the OVLT **(A)**, SFO **(B)**, and AP **(C)**, and change in MABP following microinjection of [Pyr^1^]apelin-13 (200 pmol 100 nl^–1^) in the OVLT **(D)**, SFO **(E)**, and AP **(F)**, 18 days following microinjection of either LV-scr-shRNA (light gray bar, circles) or LV-APJ-shRNA (dark gray bar, squares) simultaneously to the OVLT, SFO, and AP of WKY rats (*n* = 3/group). Data is mean ± SEM with individual responses. Data in **(A–F)** analyzed with Student’s unpaired *t*-tests, *P < 0.05, **P < 0.01. **(G)** Changes in MABP ranging from 5 days before (−5) to 18 days after microinjection (vertical dotted line) of either LV-scr-shRNA (gray line) or LV-APJ-shRNA (black line) simultaneously to the OVLT, SFO, and AP of WKY rats (*n* = 3/group). Inset bar graph shows change in MABP calculated as MABP at day -5 subtracted from MABP at day 17. **(H)** Change in LV-SBPV after microinjection of either LV-scr-shRNA (light gray bar, circles) or LV-APJ-shRNA (dark gray bar, squares), calculated as LF-SBPV at day -5 subtracted from LF-SBPV at day 17 (*n* = 3/group). **(I)** Change in MABP following hexamethonium (10 mg kg^–1^, i.v.) injection at day 16 post LV-scr-shRNA (light gray bar, circles) or LV-APJ-shRNA (dark gray bar, squares) injection (*n* = 3/group). **(J)** Representative raw traces showing changes in MABP in single animals in response to i.v. [Pyr^1^]apelin-13 (black symbols, 2 mol/kg) or saline (gray symbols) in WKY rats injected simultaneously in the OVLT, SFO, and AP with either LV-APJ-shRNA (squares) or LV-scr-shRNA (circles). Change in MAPB **(K)** and LV-SBPV **(L)** following peripheral administration of [Pyr^1^]apelin-13 (2 nmol kg^–1^) to WKY rats (*n* = 3/group) 18 days post-injection in the OVLT, SFO, and AP with either LV-scr-shRNA (light gray bar, circles) or LV-APJ-shRNA (dark gray bar, squares). Data in panels **(G–L)** is mean ± SEM with individual responses and analyzed with Student’s unpaired *t*-tests.

To elucidate whether multiple knockdown of *aplnr* expression simultaneously in the OVLT, SFO and AP in the same animal affects the depressor effect of peripherally-injected [Pyr^1^]apelin-13, WKY rats were injected with either LV-APJ-shRNA or LV-scr-shRNA in the three CVOs. qPCR analysis of *aplnr* in micropunches from the OVLT, SFO, and AP showed knockdown of *aplnr* in WKY rats injected with LV-APJ-shRNA simultaneously into the three CVOs when compared with LV-scr-shRNA-injected rats (Figures 6A–C, respectively). A fall in MABP was seen in LV-scr-shRNA-injected WKY rats following a challenge microinjection of [Pyr^1^]apelin-13 separately into the OVLT, SFO, or AP (Figures 6D–F, respectively). In comparison, the decrease in MABP did not persist in LV-APJ-shRNA-injected WKY rats (Figures 6D–F, respectively). These data confirm knockdown of *aplnr* by LV-APJ-shRNA-injection in the three regions.

Body weights did not differ between LV-APJ-shRNA- and LV-scr-shRNA-transduced WKY groups over the monitoring period (Table 1). Resting MABP (Figure 6G and insert) and LF-SBPV (Figure 6H) were unchanged in LV-APJ-shRNA-injected rats compared to LV-scr-shRNA-injected rats over the 18 day monitoring period. No change was seen in hexamethonium-induced falls in MABP between animals injected with LV-APJ-shRNA- or LV-scr-shRNA in the OVLT, SFO, and AP (Figure 6I).

Representative raw traces of MABP following administration of i.v. [Pyr^1^]apelin-13 or saline in single WKY rats with multiple CVO *aplnr* knockdowns at day 18 post-virus injection are shown in Figure 6J. Peripheral i.v injection of [Pyr^1^]apelin-13 in LV-scr-shRNA WKY rats decreased MABP (Figure 6K) and LV-SBPV (Figure 6L). The decrease in both MABP and LV-SBPV persisted in LV-APJ-shRNA-injected WKY rats despite knockdown of *aplnr* in the 3 sites (Figures 6K,L, respectively).

### Multiple CVO-Targeted *Aplnr* Knockdown Attenuates the Peripheral Depressor Effect of [Pyr^1^]apelin-13 in Hypertensive Rats

**FIGURE 7:**
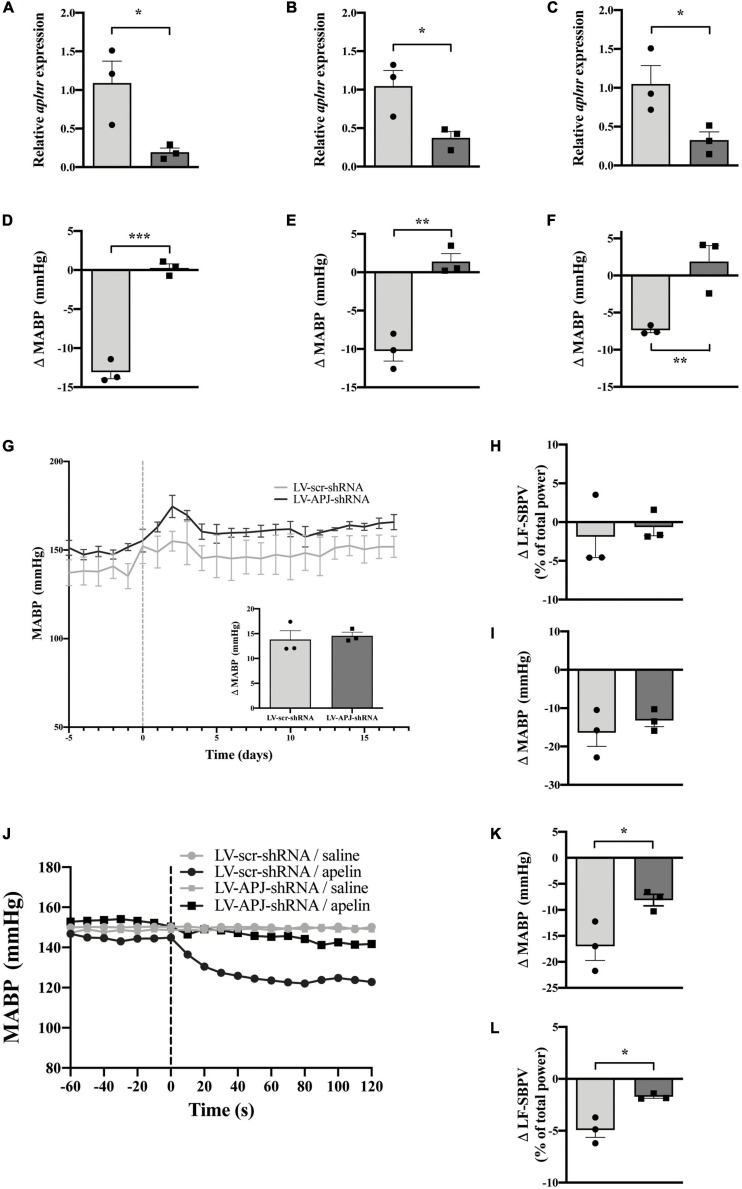
Simultaneous CVO-targeted *aplnr* knockdown attenuates the peripheral depressor effect of [Pyr^1^]apelin-13 in SHRs. Relative *aplnr* expression in the OVLT **(A)**, SFO **(B)**, and AP **(C)**, and change in MABP following microinjection of [Pyr^1^]apelin-13 (200 pmol 100 nl^–1^) in the OVLT **(D)**, SFO **(E)**, and AP **(F)**, 18 days following microinjection of either LV-scr-shRNA (light gray bar, circles) or LV-APJ-shRNA (dark gray bar, squares) simultaneously to the OVLT, SFO, and AP of SHRs (*n* = 3/group). Data is mean ± SEM with individual responses. Data in panels **(A–F)** analyzed with Student’s unpaired *t*-tests, *P < 0.05, **P < 0.01, ***P < 0.001. **(G)** Changes in MABP ranging from 5 days before (−5) to 18 days after microinjection (vertical dotted line) of either LV-scr-shRNA (gray line) or LV-APJ-shRNA (black line) simultaneously to the OVLT, SFO, and AP of SHRs (*n* = 3/group). Inset bar graph shows change in MABP calculated as MABP at day -5 subtracted from MABP at day 17. **(H)** Change in LV-SBPV after microinjection of either LV-scr-shRNA (light gray bar, circles) or LV-APJ-shRNA (dark gray bar, squares), calculated as LF-SBPV at day -5 subtracted from LF-SBPV at day 17 (*n* = 3/group). **(I)** Change in MABP following hexamethonium (10 mg kg^–1^, i.v.) injection at day 16 post LV-scr-shRNA (light gray bar, circles) or LV-APJ-shRNA (dark gray bar, squares) injection (*n* = 3/group). **(J)** Representative raw traces showing changes in MABP in single animals in response to i.v. [Pyr^1^]apelin-13 (black symbols, 2 mol kg^–1^) or saline (gray symbols) in WKY rats injected simultaneously in the OVLT, SFO, and AP with either LV-APJ-shRNA- (squares) or LV-scr-shRNA- (circles). Change in MAPB **(K)** and LV-SBPV **(L)** following peripheral administration of [Pyr^1^]apelin-13 (2 nmol/kg) to SHRs (*n* = 3/group) 18 days post-injection in the OVLT, SFO, and AP with either LV-scr-shRNA (light gray bar, circles) or LV-APJ-shRNA (dark gray bar, squares). Data in panels **(G–L)** is mean ± SEM with individual responses and analyzed with Student’s unpaired *t*-tests, *P < 0.05.

At 18-days post shRNA-injection, qPCR evaluation of micropunched OVLT, SFO, and AP tissues confirmed knockdown of *aplnr* (Figures 7A–C, respectively), and the expected decrease in MABP following microinjection of [Pyr^1^]apelin-13 onto the OVLT, SFO, and AP was prevented (Figures 7D–F, respectively) in multiple CVO-targeted LV-APJ-shRNA-injected SHRs in comparison to LV-scr-shRNA-injected SHRs.

No change was observed in MABP (Figure 7G and insert) and LF-SBPV (Figure 7H) between LV-APJ-shRNA-injected and LV-scr-shRNA-injected SHRs over the 18 day observation period after *aplnr* knockdown in all three CVO structures. Additionally, no change was seen in hexamethonium-induced falls in MABP between SHRs injected concurrently with LV-APJ-shRNA- or LV-scr-shRNA in all three sensory CVOs (Figure 7I).

Representative raw traces showing changes in MABP in response to i.v. [Pyr^1^]apelin-13 or saline administration in single SHRs simultaneously injected in the OVLT, SFO, and AP with either LV-scr-shRNA or LV-APJ-shRNA are shown (Figure 7J). Peripheral i.v injection of [Pyr^1^]apelin-13 in LV-scr-shRNA-injected SHRs decreased both MABP (Figure 7K) and LV-SBPV (Figure 7L) at day 18 post-shRNA injection. This decrease in MABP and LF-SBPV was significantly attenuated (Figures 7K,L, respectively) in SHRs with multiple CVO-targeted knockdown of *aplnr* in the same animal by LV-APJ-shRNA-injection.

## Discussion

The mechanisms by which peripheral and central apelin modulates BP remains a major question. For example, whether blood-borne apelin acts at the same locus as centrally synthesized apelin is unknown and indeed the neuronal circuitry expressing apelin is not fully understood. The present study examines the role of APJ in the sensory CVOs for regulation of arterial pressure in SHR and WKY rats. We reveal a number of novel findings. First, *aplnr* is up-regulated in the OVLT, SFO, and AP in SHRs in comparison to WKY rats. Second, microinjection of [Pyr^1^]apelin-13, acting via APJ, into any of the three CVOs decreases MABP and this is greater in the SHR. Third, knockdown of *aplnr* in the CVOs, either collectively or individually, affects neither resting MABP nor an indirect measure of sympathetic nerve activity. Fourth, *aplnr* in the three CVOs collectively modulates the acute cardiovascular responses to systemic apelin in hypertensive SHRs but not in normotensive WKY rats. In SHRs, knockdown of *aplnr* in the three sensory CVOs collectively, but not in any CVO individually, reduces both the depressor and SBPV responses to peripheral [Pyr^1^]apelin-13 administration.

Our data demonstrates that *aplnr* is expressed in the CVOs, where the lack of a BBB may facilitate the interaction of circulating apelin with APJ present in these structures. *Aplnr* is up-regulated in the OVLT, SFO, and AP of SHRs in comparison to WKY rats, consistent with our earlier study showing increased *aplnr* expression in the SFO of SHRs (Griffiths et al., 2020) and with other studies showing an up-regulated *aplnr* expression in the PVN and RVLM of SHRs (Zhang et al., 2014; Griffiths et al., 2018). It is currently unknown whether these changes in *aplnr* levels correspond to changes in levels of functional protein, however, we have shown an augmented depressor response to microinjection of [Pyr^1^]apelin-13 in the SHR CVOs in comparison to WKY rats, that supports raised APJ expression in the SHR. The factors that drive increased *aplnr* expression in the SHR CVOs are not known. The up-regulation in gene expression may be compensatory and contribute to the effect of apelin given peripherally. Such compensatory changes [e.g., remodeling of microvascular and glial cells (Ritz et al., 2009) with possible alterations of *aplnr* levels in these cells] may have consequences for the activity of CVO efferent projections, and may help modulate BP in the face of hypertension. It is unknown whether the higher resting sympathetic tone of SHRs (Iriuchijima, 1973; Judy et al., 1976) contributes to the more pronounced BP responses to CVO [Pyr^1^]apelin-13 microinjection observed in SHRs. Apelinergic gene expression levels are altered in conditions of hypertension, e.g., myocardium *aplnr*/*apln* levels are down-regulated in experimental heart failure in Dahl salt-sensitive rats (Iwanaga et al., 2006) and decreased *aplnr*/*apln* levels are seen in the heart and aorta of SHRs compared to normotensive rats (Zhong et al., 2005), while *aplnr*/*apln* levels and apelin/APJ protein are up-regulated in the PVN of SHR (Zhang et al., 2014), reflecting a differential peripheral vs. central expression pattern. Additionally, circulating levels of apelin are decreased in chronic heart failure (Földes et al., 2003) and in essential and pulmonary hypertension (Sonmez et al., 2010; Chandra et al., 2011), and increased in disease states such as aortic stenosis (Duman et al., 2018), and left ventricular hypertrophy (Helske et al., 2010). These findings highlight the importance of understanding the physiological and pathophysiological roles of central and peripheral apelin in cardiovascular control.

We have previously shown our APJ-specific shRNA viral vector is successful in *in vivo* knockdown of central *aplnr* in the rat RVLM (Griffiths et al., 2018), with transgene expression highly expressed 3 weeks after transfection. Using the same construct in the present study, we established CVO knockdown of *aplnr* in all LV-APJ-shRNA-injected rats. This was confirmed both by qPCR analysis showing efficient LV-APJ-shRNA-induced suppression of CVO *aplnr* expression and also by subsequent loss of functionality, i.e., loss of APJ CVO depressor response to apelin *in vivo* after microinjection of exogenous [Pyr^1^]apelin-13 into the CVO, in knockdown animals, demonstrating robust and reliable reduction of central apelin-mediated cardiovascular effects in the CVOs by LV-APJ-shRNA.

To investigate the role of *aplnr* in specific sensory CVOs as potential sites influencing the effects of central and/or peripheral apelin on BP regulation, we initially targeted knockdown of *aplnr* in individual CVOs in both SHRs and WKY rats. We show that chronic decreased *aplnr* expression in any individual CVO after LV-APJ-shRNA microinjection did not result in a decrease of MABP, or in sympathoinhibition (measured indirectly using power spectral analysis and hexamethonium), in normotensive or hypertensive rats when compared to control LV-scr-shRNA-injected rats. We also investigated the effect of *aplnr* knockdown on the cardiovascular responses to peripherally administered apelin. This protocol represents physiological conditions, where injected apelin has access to peripheral APJ and to receptors present in the CVOs as well, perhaps, in other brain regions—e.g., at the BBB where APJ is expressed in vascular cells and astrocytes. Apelin injected i.p. has been shown to increase hypothalamic (Higuchi et al., 2007) and cerebrospinal fluid (Sinen and Bülbül, 2021) apelin levels, and to activate c-Fos in the PVN (Takayama et al., 2008) and CVO (Sinen and Bülbül, 2021), indicating that systemic apelin may be able to cross the BBB to stimulate central neurons. Based on studies of many peptides it is unlikely that polar substances such as apelin-13 and larger apelin fragments would cross the BBB by diffusion but may enter the brain parenchyma via carrier-mediated and/or receptor mediated transport (Lee and Jayant, 2019). We demonstrate that 18 days after LV-APJ-shRNA injection into individual CVOs, the decrease in BP and LF-SBPV observed following peripheral administration of [Pyr^1^]apelin-13 was maintained despite *aplnr* knockdown in the CVO. Our results suggest that endogenous APJ activity in individual CVOs has no effect on basal control of BP and that silencing of *aplnr* in the OVLT, SFO or AP individually does not modify the peripheral depressor effect of [Pyr^1^]apelin-13.

To clarify the role of *aplnr* in the CVOs on BP regulation, we then investigated the BP effects of chronic *aplnr* knockdown in three CVOs simultaneously in normotensive and hypertensive rats. Knockdown of *aplnr* in three CVOs in the same animals did not have a significant effect on BP or on indirect measure of sympathoinhibition monitored over 18 days, indicating an absence of endogenous apelin-mediated cardiovascular regulation. In contrast to tonic control of BP, we report acute effects on both MABP and indirectly measured sympathetic activity in response to exogenous application of [Pyr^1^]apelin-13 after simultaneous CVO *aplnr* knockdown in SHRs. In SHRs, but not in WKY rats, 18 days after *aplnr*-specific virus injection, simultaneous knockdown of *aplnr* in three CVO structures caused an immediate attenuation in the depressor effect, and in LF-SBPV, normally seen in response to peripherally administered [Pyr^1^]apelin-13. This suggests that functional APJ is required in the CVOs for an intact cardiovascular response to peripherally administered apelin in the hypertensive animal model used in this study. Thus, it appears that in physiological conditions apelin does not act in the CVOs to maintain baseline MABP, but when circulating apelin is increased the integrity of *aplnr* in the CVOs may be essential for a conventional BP response to apelin. An increase of apelin in the circulation, acting upon increased levels of APJ in the CVOs, may regulate sympathetic outflow by stimulating descending neural pathways, additionally offering therapeutic potential for APJ agonism in pathophysiological conditions. It is important to note that the central circuitry of APJ-expressing cell bodies and APJ-projecting fibers in either rodents or humans has not been detailed.

In our study, if APJ in one of the sensory CVOs was solely responsible for responding to changing levels of circulating apelin and transmitting this information to autonomic control centers to regulate BP, then knockdown of *aplnr* in that discrete CVO should have resulted in a change in the BP response to peripherally administered [Pyr^1^]-apelin-13. The sensory CVOs are known to operate in a system of functional redundancy (McKinley et al., 2019) and of reciprocity of communication with regions of the cerebral cortex that regulate autonomic function (Menani et al., 2014), and so it is reasonable to speculate that the loss of functional APJ integrity in one CVO may be compensated for by the remaining CVOs. By contrast, SHRs with *aplnr* CVO knockdown in three CVOs exhibited a significant deficit in the depressor response to peripheral [Pyr^1^]apelin-13, and in an interesting corollary, this deficit is linked to an attenuation of the spectral activity response in these rats, an indirect marker of sympathetic tone. Our findings indicate that multiple CVO *aplnr* knockdown eliminates redundancy in these central structures. The sensory CVOs are not the sole sites of action through which circulating apelin modulates pathways involved in BP regulation as silencing of CVO APJ activity did not completely abolish BP and LV-SBPV responses to [Pyr^1^]apelin-13 administration; however, our data indicates that the CVOs play key roles in this process. To our knowledge the combinatory importance of the sensory CVOs in the cardiovascular response to other peripherally administered neuropeptides *in vivo* has not been reported.

CVOs such as the SFO, OVLT, and AP exhibit considerable transcriptomic plasticity in response to experimental manipulations including thirst (Hindmarch et al., 2008, 2011; Pool et al., 2020), food deprivation (Hindmarch et al., 2008), or early life overnutrition (Peterson et al., 2018). Some of this plasticity is contributed by vascular elements (Morita et al., 2015). *Aplnr* is expressed in neurons, glia and vascular endothelial cells in the brain (Zhang et al., 2016; Griffiths et al., 2020). Gene-targeted [e.g., single-cell fluorescence *in situ* hybridization (FISH)] or global (e.g., single-cell RNAseq) transcriptomic studies will allow the assessment of the contribution of different *aplnr*-expressing CVO cell types in response to elevated BP. Such studies will also shed light on whether APJ potentially interacts with other receptors in the CVOs, most notably ones, like the angiotensin AT1 receptor, with which it has been shown to heterodimerize *in vitro* (Chun et al., 2008).

In summary, we have shown a significant role for APJ in the CVOs in the regulation of acute cardiovascular responses to systemic apelin in SHRs. Importantly, this study has demonstrated the functional significance of APJ in the SFO, OVLT, and AP collectively as a primary feedback interface between circulating apelin and critical cardiovascular control centers that drive SNA activity to regulate BP. Central apelinergic pathways may be recruited to buffer the peripheral effects of apelin, working in a coordinated fashion to maintain BP within normal physiological limits. Understanding the central circuitry through which systemic apelin affects central cardiovascular responses will increase our understanding of the pathogenesis of hypertension and aid in elucidating the therapeutic potential of the apelinergic pathway in a hypertensive disease state.

## Data Availability Statement

The original contributions presented in the study are included in the article/supplementary material, further inquiries can be directed to the corresponding author/s.

## Ethics Statement

The animal study was reviewed and approved by the University of Bristol Animal Welfare and Ethical Review Body.

## Author Contributions

A-MO’C, SL, and JP were responsible for acquisition of funding. A-MO’C, PG, JP, and SL contributed to the conception, design of the research, edited, and revised the manuscript. PG performed experiments. A-MO’C, SL, and PG interpreted results of the experiments. A-MO’C and PG drafted the manuscript. All authors have approved the final version of the manuscript and agreed to be accountable for all aspects of the work. All persons designated as authors qualify for authorship, and all those who qualify for authorship are listed.

## Conflict of Interest

The authors declare that the research was conducted in the absence of any commercial or financial relationships that could be construed as a potential conflict of interest.

## Publisher’s Note

All claims expressed in this article are solely those of the authors and do not necessarily represent those of their affiliated organizations, or those of the publisher, the editors and the reviewers. Any product that may be evaluated in this article, or claim that may be made by its manufacturer, is not guaranteed or endorsed by the publisher.

## References

[B1] Barnes G. Japp A. G. Newby D. E. (2010). Translational promise of the apelin-APJ system. *Heart* 96 1011–1016. 10.1136/hrt.2009 20584856

[B2] Boucher J. Masri B. Daviaud D. Gesta S. Guigné C. Mazzucotelli A. (2005). Apelin, a newly identified adipokine up-regulated by insulin and obesity. *Endocrinology* 146 1764–1771. 10.1210/en.2004-1427 15677759

[B3] Carretero O. A. Oparil S. (2000). Essential hypertension. Part I: definition and etiology. *Circulation* 101 329–335. 10.1161/01.cir.101.3.329 10645931

[B4] Cavallo M. G. Sentinelli F. Barchetta I. Costantino C. Incani M. Perra L. (2012). Altered glucose homeostasis is associated with increased serum apelin levels in type 2 diabetes mellitus. *PLoS One* 7:e51236. 10.1371/journal.pone.0051236 23227256 PMC3515542

[B5] Chandra S. M. Razavi H. Kim J. Agrawal R. Kundu R. K. de Jesus Perez V. (2011). Disruption of the apelin-APJ system worsens hypoxia-induced pulmonary hypertension. *Arterioscler. Thromb. Vasc. Biol.* 31 814–820. 10.1161/ATVBAHA.110.219980 21233449 PMC3113525

[B6] Chun H. J. Ali Z. A. Kojima Y. Kundu R. K. Sheikh A. Y. Agrawal R. (2008). Apelin signaling antagonizes AngII effects in mouse models of atherosclerosis. *J. Clin. Invest*. 118 3343–3354. 10.1172/JCI34871 18769630 PMC2525695

[B7] Cottrell G. T. Ferguson A. V. (2004). Sensory circumventricular organs: central roles in integrated autonomic regulation. *Regul. Pept.* 117 11–23. 10.1016/j.regpep.2003.09.004 14687696

[B8] Dai L. Smith P. M. Kuksis M. Ferguson A. V. (2013). Apelin acts in the subfornical organ to influence neuronal excitability and cardiovascular function. *J. Physiol.* 591 3421–3432. 10.1113/jphysiol.2013.254144 23629509 PMC3717236

[B9] De Mota N. Reaux-Le Goazigo A. El Messari S. Chartrel N. Roesch D. Dujardin C. (2004). Apelin, a potent diuretic neuropeptide counteracting vasopressin actions through inhibition of vasopressin neuron activity and vasopressin release. *Proc. Natl. Acad. Sci. U.S.A*. 101 10464–10469. 10.1073/pnas.0403518101 15231996 PMC478592

[B10] Dray C. Debard C. Jager J. Disse E. Daviaud D. Martin P. (2010). Apelin and APJ regulation in adipose tissue and skeletal muscle of type 2 diabetic mice and humans. *Am. J. Physiol. Endocrinol. Metab.* 298 E1161–E1169. 10.1152/ajpendo.00598.2009 20233941

[B11] Dray C. Knauf C. Daviaud D. Waget A. Boucher J. Buléon M. (2008). Apelin stimulates glucose utilization in normal and obese insulin-resistant mice. *Cell Metab.* 8 437–445. 10.1016/j.cmet.2008.10.003 19046574

[B12] Duman H. Bahçeci I. Hamur H. Demirelli S. Ramazan Dilek A. Erdogan T. (2018). The relationship between serum apelin levels and the severity of calcific aortic stenosis. *Acta Cardiol. Sin.* 34 259–266. 10.6515/ACS.201805_34(3).20180207A 29844647 PMC5968342

[B13] Ferdinal F. Limanan D. Rini R. D. Alexsandro R. Helmi R. (2019). Elevated levels of apelin-36 in heart failure due to chronic systemic hypoxia. *Int. J. Angiol.* 28 194–199. 10.1055/s-0038-1676340 31452587 PMC6707794

[B14] Fisher J. P. Paton J. F. R. (2012). The sympathetic nervous system and blood pressure in humans: implications for hypertension. *J. Hum. Hypertens.* 26 463–475. 10.1038/jhh.2011.66 21734720

[B15] Földes G. Horkay F. Szokodi I. Vuolteenaho O. Ilves M. Lindstedt K. A. (2003). Circulating and cardiac levels of apelin, the novel ligand of the orphan receptor APJ, in patients with heart failure. *Biochem. Biophys. Res. Commun.* 308 480–485. 10.1016/s0006-291x(03)01424-4 12914775

[B16] Griffiths P. R. Lolait S. J. Bijabhai A. O’Carroll-Lolait A. Paton J. F. R. O’Carroll A.-M. (2020). Increased apelin receptor gene expression in the subfornical organ of spontaneously hypertensive rats. *PLoS One* 15:e0231844. 10.1371/journal.pone.0231844 32315363 PMC7173921

[B17] Griffiths P. R. Lolait S. J. Pearce L. E. McBryde F. D. Paton J. F. R. O’Carroll A.-M. (2018). Blockade of rostral ventrolateral medulla apelin receptors does not attenuate arterial pressure in SHR and _L_-NAME-induced hypertensive rats. *Front. Physiol.* 9:1488. 10.3389/fphys.2018.01488 30459635 PMC6232890

[B18] Hasser E. M. Cunningham J. T. Sullivan M. J. Curtis K. S. Blaine E. H. Hay M. (2000). Area postrema and sympathetic nervous system effects of vasopressin and angiotensin II. *Clin. Exp. Pharmacol. Physiol.* 27 432–436. 10.1046/j.1440-1681.2000.03261.x 10831249

[B19] Hatzelmann T. Ott D. Marks D. Gerstberger R. (2009). Functional expression of the apelin-12 receptor protein APJ in rat hypothalamic nuclei (PVN and MnPO) involved in body fluid homeostasis and temperature regulation. *Acta Physiol.* 195 (Suppl. 669), 460.

[B20] Helske S. Kovanen P. T. Lommi J. Turto H. Kupari M. (2010). Transcardiac gradients of circulating apelin: extraction by normal hearts vs. release by hearts failing due to pressure overload. *J. Appl. Physiol.* 109 1744–1748. 10.1152/japplphysiol.00474.2010 20864562

[B21] Higuchi K. Masaki T. Gotoh K. Chiba S. Katsuragi I. Tanaka K. (2007). Apelin, an APJ receptor ligand, regulates body adiposity and favors the messenger ribonucleic acid expression of uncoupling proteins in mice. *Endocrinology* 148 2690–2697. 10.1210/en.2006-1270 17347313

[B22] Hindmarch C. C. Fry M. Smith P. M. Yao S. T. Hazell G. G. J. Lolait S. J. (2011). The transcriptome of the medullary area postrema: the thirsty rat, the hungry rat and the hypertensive rat. *Exp. Physiol.* 96 495–504. 10.1113/expphysiol.2010.056515 21317217

[B23] Hindmarch C. Fry M. Yao S. T. Smith P. M. Murphy D. Ferguson A. V. (2008). Microarray analysis of the transcriptome of the subfornical organ in the rat: regulation by fluid and food deprivation. *Am. J. Physiol. Regul. Integr. Comp. Physiol.* 295 R1914–R1920. 10.1152/ajpregu.90560.2008 18832082

[B24] Iriuchijima J. (1973). Sympathetic discharge rate in spontaneously hypertensive rats. *Jpn. Heart J.* 14 350–356. 10.1536/ihj.14.350 4542949

[B25] Ishida J. Hashimoto T. Hashimoto Y. Nishiwaki S. Iguchi T. Harada S. (2004). Regulatory roles for APJ, a seven-transmembrane receptor related to angiotensin-type 1 receptor in blood pressure in vivo. *J. Biol. Chem.* 279 26274–26279. 10.1074/jbc.M404149200 15087458

[B26] Iwanaga Y. Kihara Y. Takenaka H. Kita T. (2006). Down-regulation of cardiac apelin system in hypertrophied and failing hearts: possible role of angiotensin II-angiotensin type 1 receptor system. *J. Mol. Cell Cardiol.* 41 798–806. 10.1016/j.yjmcc.2006.07.004 16919293

[B27] Japundžić-Žigon N. (1998). Physiological mechanisms in regulation of blood pressure fast frequency variations. *Clin. Exp. Hypertens.* 20 359–388. 10.3109/10641969809053219 9607401

[B28] Judy W. V. Watanabe A. M. Henry D. P. Besch H. R. Jr. Murphy W. R. Hockel G. M. (1976). Sympathetic nerve activity: role in regulation of blood pressure in the spontaenously hypertensive rat. *Circ. Res.* 38 21–29. 10.1161/01.res.38.6.21 178466

[B29] Kang Y. Kim J. Anderson J. P. Wu J. Gleim S. R. Kundu R. K. (2013). Apelin-APJ signaling is a critical regulator of endothelial MEF2 activation in cardiovascular development. *Circ. Res.* 113 22–31. 10.1161/CIRCRESAHA.113.301324 23603510 PMC3739451

[B30] Kuba K. Zhang L. Imai Y. Arab S. Chen M. Maekawa Y. (2007). Impaired heart contractility in Apelin gene-deficient mice associated with aging and pressure overload. *Circ. Res.* 101 e32–e42. 10.1161/CIRCRESAHA.107.158659 17673668

[B31] Lee D. K. Cheng R. Nguyen T. Fan T. Kariyawasam A. P. Liu Y. (2000). Characterisation of apelin, the ligand for the APJ receptor. *J. Neurochem.* 74 34–41. 10.1046/j.1471-4159.2000.0740034.x 10617103

[B32] Lee D. K. Saldivia V. R. Nguyen T. Cheng R. George S. R. O’Dowd B. F. (2005). Modification of the terminal residue of apelin-13 antagonizes its hypotensive action. *Endocrinology* 146 231–236. 10.1210/en.2004-0359 15486224

[B33] Lee M. R. Jayant R. D. (2019). Penetration of the blood-brain barrier by peripheral neuropeptides: new approaches to enhancing transport and endogenous expression. *Cell Tiss. Res.* 375 287–293. 10.1007/s00441-018-2959-y 30535799 PMC6467522

[B34] Li L. Yang G. Li Q. Tang Y. Yang M. Yang H. (2006). Changes and relations of circulating visfatin, apelin, and resistin levels in normal, impaired glucose tolerance, and type 2 diabetic subjects. *Exp. Clin. Endocrinol. Diabetes* 114 544–548. 10.1055/s-2006-948309 17177135

[B35] Livak K. J. Schmittgen T. D. (2001). Analysis of relative gene expression data using real-time quantitative PCR and the 2(−Delta Delta C(T)) Method. *Methods* 25 402–408. 10.1006/meth.2001.1262 11846609

[B36] Lozić M. Greenwood M. Šarenac O. Martin A. Hindmarch C. Tasić T. (2014). Overexpression of oxytocin receptors in the hypothalamic PVN increases baroreceptor reflex sensitivity and buffers BP variability in conscious rats. *Br. J. Pharmacol.* 171 4385–4398. 10.1111/bph.12776 24834854 PMC4209146

[B37] Maguire J. J. Kleinz M. J. Pitkin S. L. Davenport A. P. (2009). [Pyr^1^]apelin-13 identified as the predominant apelin isoform in the human heart: vasoactive mechanisms and inotropic action in disease. *Hypertension* 54 598–604. 10.1161/HYPERTENSIONAHA.109.134619 19597036

[B38] McBryde F. D. Abdala A. P. Hendy E. B. Pijacka W. Marvar P. Moraes D. J. (2013). The carotid body as a putative therapeutic target for the treatment of neurogenic hypertension. *Nat. Commun.* 4 2395–2405. 10.1038/ncomms3395 24002774

[B39] McKinley M. J. Allen A. M. May C. N. McAllen R. M. Oldfield B. J. Sly D. (2001). Neural pathways from the lamina terminalis influencing cardiovascular and body fluid homeostasis. *Clin. Exp. Pharmacol. Physiol.* 28 990–992. 10.1046/j.1440-1681.2001.03592.x 11903300

[B40] McKinley M. J. Denton D. A. Ryan P. J. Yao S. T. Stefanidis A. Oldfield B. J. (2019). From sensory circumventricular organs to cerebral cortex: neural pathways controlling thirst and hunger. *J. Neuroendocrinol.* 31:e12689. 10.1111/jne.12689 30672620

[B41] Menani J. V. Vieira A. A. Colombari D. S. A. De Paula P. M. Colombari E. De Luca L. A. Jr. (2014). “Preoptic–periventricular integrative mechanisms involved in behavior, fluid–electrolyte balance, and pressor responses,” in *Neurobiology of Body Fluid Homeostasis: Transduction and Integration*, eds Menani J. V. Johnson A. K. (Boca Raton, FL: CRC Press).

[B42] Mitra A. Katovich M. J. Mecca A. Rowland N. E. (2006). Effects of central and peripheral injections of apelin on fluid intake and cardiovascular parameters in rats. *Physiol. Behav.* 89 221–225. 10.1016/j.physbeh.2006.06.006 16839572

[B43] Morita S. Furube E. Mannari T. Okuda H. Tatsumi K. Wanaka A. (2015). Vascular endothelial growth factor-dependent angiogenesis and dynamic vascular plasticity in the sensory circumventricular organs of the adult mouse brain. *Cell Tissue Res*. 359 865–884. 10.1007/s00441-014-2080-9 25573819

[B44] O’Carroll A.-M. Lolait S. J. (2003). Regulation of rat APJ receptor messenger ribonucleic acid expression in magnocellular neurones of the paraventricular and supraoptic nuclei by osmotic stimuli. *J. Neuroendocrinol.* 15 661–666. 10.1046/j.1365-2826.2003.01044.x 12787050

[B45] O’Carroll A.-M. Don A. L. Lolait S. J. (2003). APJ receptor mRNA expression in the rat hypothalamic paraventricular nucleus: regulation by stress and glucocorticoids. *J. Neuroendocrinol.* 15 1095–1101. 10.1046/j.1365-2826.2003.01102.x 14622440

[B46] O’Carroll A.-M. Lolait S. J. Harris L. E. Pope G. R. (2013). The apelin receptor APJ: journey from an orphan to a multifaceted regulator of homeostasis. *J. Endocrinol.* 219 R13–R35. 10.1530/JOE-13-0227 23943882

[B47] O’Dowd B. F. Heiber M. Chan A. Heng H. H. Tsui L. C. Kennedy J. L. (1993). A human gene that shows identity with the gene encoding the angiotensin receptor is located on chromosome 11. *Gene* 136 355–360. 10.1016/0378-1119(93)90495-o 8294032

[B48] O’Harte F. P. M. Parthsarathy V. Hogg C. Flatt P. R. (2018). Long-term treatment with acylated analogues of apelin-13 amide ameliorates diabetes and improves lipid profile of high-fat fed mice. *PLoS One* 13:e0202350. 10.1371/journal.pone.0202350 30157220 PMC6114795

[B49] Parati G. Saul J. P. Di Rienzo M. Mancia G. (1995). Spectral analysis of blood pressure and heart rate variability in evaluating cardiovascular regulation. a critical appraisal. *Hypertension* 25 1276–1286. 10.1161/01.hyp.25.6.1276 7768574

[B50] Parthsarathy V. Hogg C. Flatt P. R. O’Harte F. P. M. (2018). Beneficial long-term antidiabetic actions of N- and C-terminally modified analogues of apelin-13 in diet-induced obese diabetic mice. *Diabetes Obes. Metab.* 20 319–327. 10.1111/dom.13068 28730728

[B51] Paxinos G. Watson C. (2005). *The Rat Brain in Stereotaxic Coordinates*, Compact 6th Edn. New York, NY: Academic Press.

[B52] Peterson C. S. Huang S. Lee S. A. Ferguson A. V. Fry W. M. (2018). The transcriptome of the rat subfornical organ is altered in response to early postnatal overnutrition. *IBRO Rep.* 5 17–23. 10.1016/j.ibror.2018.06.001 30135952 PMC6095096

[B53] Pool A.-H. Wang T. Stafford D. A. Chance R. K. Lee S. Ngai J. (2020). The cellular basis of distinct thirst modalities. *Nature* 588 112–117. 10.1038/s41586-020-2821-8 33057193 PMC7718410

[B54] Reaux A. De Mota N. Skultetyova I. Lenkei Z. El Messari S. Gallatz K. (2001). Physiological role of a novel neuropeptide, apelin, and its receptor in the rat brain. *J. Neurochem.* 77 1085–1096. 10.1046/j.1471-4159.2001.00320.x 11359874

[B55] Reaux-Le Goazigo A. Bodineau L. De Mota N. Jeandel L. Chartrel N. Knauf C. (2011). Apelin and the proopiomelanocortin system: a new regulatory pathway of hypothalamic α-MSH release. *Am. J. Physiol. Endocrinol. Metab.* 301 E955–E966. 10.1152/ajpendo.00090.2011 21846903

[B56] Ritz M.-F. Fluri F. Engelter S. T. Schaeren-Wiemers N. Lyrer P. A. (2009). Cortical and putamen age-related changes in the microvessel density and astrocyte deficiency in spontaneously hypertensive and stroke-prone spontaneously hypertensive rats. *Curr. Neurovasc. Res*. 6 279–287. 10.2174/156720209789630311 19807651

[B57] Ronkainen V. P. Ronkainen J. J. Hänninen S. L. Leskinen H. Ruas J. L. Pereira T. (2007). Hypoxia inducible factor regulates the cardiac expression and secretion of apelin. *FASEB J*. 21 1821–1830. 10.1096/fj.06-7294com 17341685

[B58] Sato T. Kadowaki A. Suzuki T. Ito H. Watanabe H. Imai Y. (2019). Loss of apelin augments angiotensin II-induced cardiac dysfunction and pathological remodeling. *Int. J. Mol. Sci.* 20:239. 10.3390/ijms20020239 30634441 PMC6358887

[B59] Sawane M. Kajiya K. Kidoya H. Takagi M. Muramatsu F. Takakura N. (2013). Apelin inhibits diet-induced obesity by enhancing lymphatic and blood vessel integrity. *Diabetes* 62 1970–1980. 10.2337/db12-0604 23378608 PMC3661640

[B60] Seyedabadi M. Goodchild A. K. Pilowsky P. M. (2002). Site-specific effects of apelin-13 in the rat medulla oblongata on arterial pressure and respiration. *Auton. Neurosci.* 101 32–38. 10.1016/s1566-0702(02)00178-9 12462357

[B61] Siddiquee K. Hampton J. McAnally D. May L. Smith L. (2013). The apelin receptor inhibits the angiotensin II type 1 receptor via allosteric trans-inhibition. *Br. J. Pharmacol.* 168 1104–1117. 10.1111/j.1476-5381.2012.02192.x 22935142 PMC3594671

[B62] Sinen O. Bülbül M. (2021). The role of autonomic pathways in peripheral apelin-induced gastrointestinal dysmotility: involvement of the circumventricular organs. *Exp. Physiol*. 106 475–485. 10.1113/EP089182 33347671

[B63] Smith P. M. Ferguson A. V. (1997). Vasopressin acts in the subfornical organ to decrease blood pressure. *Neuroendocrinology* 66 130–135. 10.1159/000127230 9263210

[B64] Smith P. M. Chambers A. P. Price C. J. Ho W. Hopf C. Sharkey K. A. (2009). The subfornical organ: a central nervous system site for actions of circulating leptin. *Am. J. Physiol. Regul. Integr. Comp. Physiol.* 296 R512–R520. 10.1152/ajpregu.90858.2008 19020290

[B65] Sonmez A. Celebim G. Erdem G. Tapan S. Genc H. Tasci I. (2010). Plasma apelin and ADMA levels in patients with essential hypertension. *Clin. Exp. Hypertens.* 32 179–183. 10.3109/10641960903254505 20504125

[B66] Sörhede Winzell M. Magnusson C. Ahrén B. (2005). The apj receptor is expressed in pancreatic islets and its ligand, apelin, inhibits insulin secretion in mice. *Regul. Pept.* 131 12–17. 10.1016/j.regpep.2005.05.004 15970338

[B67] Soriguer F. Garrido-Sanchez L. Garcia-Serrano S. Garcia-Almeida J. M. Garcia-Arnes J. Tinahones F. J. (2009). Apelin levels are increased in morbidly obese subjects with Type 2 Diabetes Mellitus. *Obes. Surg.* 19 1574–1580. 10.1007/s11695-009-9955-y 19756893

[B68] Sunn N. McKinley M. J. Oldfield B. J. (2003). Circulating angiotensin II activates neurones in circumventricular organs of the lamina terminalis that project to the bed nucleus of the stria terminalis. *J. Neuroendocrinol.* 15 725–731. 10.1046/j.1365-2826.2003.00969.x 12834432

[B69] Swanson L. W. Kuypers H. G. (1980). The paraventricular nucleus of the hypothalamus: cytoarchitectonic subdivisions and organization of projections to the pituitary, dorsal vagal complex, and spinal cord as demonstrated by retrograde fluorescence double-labeling methods. *J. Comp. Neurol* 194 555–570. 10.1002/cne.901940306 7451682

[B70] Takayama K. Iwazaki H. Hirabayashi M. Yakabi K. Ro S. (2008). Distribution of c-Fos immunoreactive neurons in the brain after intraperitoneal injection of apelin-12 in Wistar rats. *Neurosci. Lett.* 43 247–250. 10.1016/j.neulet.2007.11.048 18164129

[B71] Tatemoto K. Hosoya M. Habata Y. Fujii R. Kakegawa T. Zou M. X. (1998). Isolation and characterization of a novel endogenous peptide ligand for the human APJ receptor. *Biochem. Biophys. Res. Commun.* 251 471–476. 10.1006/bbrc.1998.9489 9792798

[B72] Trippodo N. C. Frohlich E. D. (1981). Similarities of genetic (spontaneous) hypertension. Man and rat. *Circ. Res.* 48 309–319. 10.1161/01.res.48.3.309 7460205

[B73] Wadei H. M. Textor S. C. (2012). The role of the kidney in regulating arterial blood pressure. *Nat. Rev. Nephrol.* 8 602–609. 10.1038/nrneph.2012.191 22926246

[B74] Waki H. Katahira K. Polson J. W. Kasparov S. Murphy D. Paton J. F. (2006). Automation of analysis of cardiovascular autonomic function from chronic measurements of arterial pressure in conscious rats. *Exp. Physiol.* 91 201–213. 10.1113/expphysiol.2005.031716 16239254

[B75] Wang Z. Yu D. Wang M. Wang Q. Kouznetsova J. Yang R. (2015). Elabela-apelin receptor signaling pathway is functional in mammalian systems. *Sci. Rep.* 5:8170. 10.1038/srep08170 25639753 PMC4313117

[B76] Yang P. Read C. Kuc R. E. Buonincontri G. Southwood M. Torella R. (2017). Elabela/Toddler is an endogenous agonist of the apelin APJ receptor in the adult cardiovascular system, and exogenous administration of the peptide compensates for the downregulation of its expression in pulmonary arterial hypertension. *Circulation* 135 1160–1173. 10.1161/CIRCULATIONAHA.116.023218 28137936 PMC5363837

[B77] Zhang F. Sun H.-J. Xiong X.-Q. Chen Q. Li Y.-H. Kang Y.-M. (2014). Apelin-13 and APJ in paraventricular nucleus contribute to hypertension via sympathetic activation and vasopressin release in SHR. *Acta Physiol.* 212 17–27. 10.1111/apha.12342 24995933

[B78] Zhang J. Ren C. X. Qi Y. F. Lou L. X. Chen L. Zhang L. K. (2006). Exercise training promotes expression of apelin and APJ of cardiovascular tissues in spontaneously hypertensive rats. *Life Sci.* 79 1153–1159. 10.1016/j.lfs.2006.03.040 16674982

[B79] Zhang Q. Yao F. Raizada M. K. O’Rourke S. T. Sun C. (2009). Apelin gene transfer into the rostral ventrolateral medulla induces chronic blood pressure elevation in normotensive rats. *Circ. Res.* 104 1421–1428. 10.1161/CIRCRESAHA.108.192302 19443838 PMC2746457

[B80] Zhang Y. Sloan S. Clarke L. E. Caneda C. Plaza C. A. Blumenthal P. D. (2016). Purification and characterization of progenitor and mature human astrocytes reveals transcriptional and functional differences with mouse. *Neuron* 89 37–53. 10.1016/j.neuron.2015.11.013 26687838 PMC4707064

[B81] Zhong J. C. Huang D. Y. Liu G. F. Jin H. Y. Yang Y. M. Li Y. F. (2005). Effects of all-trans retinoic acid on orphan receptor APJ signaling in spontaneously hypertensive rats. *Cardiovasc. Res.* 65 743–750. 10.1016/j.cardiores.2004.10.020 15664402

[B82] Zhu P. Huang F. Lin F. Yuan Y. Chen F. Li Q. (2013). Plasma apelin levels, blood pressure and cardiovascular risk factors in a coastal Chinese population. *Ann. Med.* 45 494–498. 10.3109/07853890.2013.833767 24032577

[B83] Zubcevic J. Waki H. Raizada M. K. Paton J. F. (2011). Autonomic-immune-vascular interaction: an emerging concept for neurogenic hypertension. *Hypertension* 57 1026–1033. 10.1161/HYPERTENSIONAHA.111.169748 21536990 PMC3105900

